# General practitioners' experiences with sickness certification: a comparison of survey data from Sweden and Norway

**DOI:** 10.1186/1471-2296-13-10

**Published:** 2012-03-01

**Authors:** Lee D Winde, Kristina Alexanderson, Benedicte Carlsen, Linnea Kjeldgård, Anna Löfgren Wilteus, Sturla Gjesdal

**Affiliations:** 1 Department of Public Health and Primary Health Care, Faculty of Medicine and Dentistry, University of Bergen, Bergen, Norway; 2 Division of Insurance Medicine, Department of Clinical neuroscience, Karolinska Institutet, Stockholm, Sweden; 3 Uni Rokkan Centre, Bergen, Norway; 4 Division of Neuropediatrics, Department of Women's and Children's Health, Karolinska Institutet, Stockholm, Sweden

## Abstract

**Background:**

In most countries with sickness insurance systems, general practitioners (GPs) play a key role in the sickness-absence process. Previous studies have indicated that GPs experience several tasks and situations related to sickness certification consultations as problematic. The fact that the organization of primary health care and social insurance systems differ between countries may influence both GPs' experiences and certification. The aim of the present study was to gain more knowledge of GPs' experiences of sickness certification, by comparing data from Sweden and Norway, regarding frequencies and aspects of sickness certification found to be problematic.

**Methods:**

Statistical analyses of cross-sectional survey data of sickness certification by GPs in Sweden and Norway. In Sweden, all GPs were included, with 3949 (60.6%) responding. In Norway, a representative sample of GPs was included, with 221 (66.5%) responding.

**Results:**

Most GPs reported having consultations involving sickness certification at least once a week; 95% of the GPs in Sweden and 99% of the GPs in Norway. A majority found such tasks problematic; 60% of the GPs in Sweden and 53% in Norway. In a logistic regression, having a higher frequency of sickness certification consultations was associated with a higher risk of experiencing them as problematic, in both countries. A higher rate of GPs in Sweden than in Norway reported meeting patients wanting a sickness certification without a medical reason. GPs in Sweden found it more problematic to discuss the advantages and disadvantages of sick leave with patients and to issue a prolongation of a sick-leave period initiated by another physician. GPs in Norway more often worried that patients would go to another physician if they did not issue a certificate, and a higher proportion of Norwegian GPs found it problematic to handle situations where they and their patient disagreed on the need for sick leave.

**Conclusions:**

The study confirms that many GPs experience sickness absence consultations as problematic. However, there were differences between the two countries in GPs' experiences, which may be linked to differences in social security regulations and the organization of GP services. Possible causes and consequences of national differences should be addressed in future studies.

## Background

In most countries with social insurance systems, general practitioners (GPs) play a key role in the sickness-absence process. Nevertheless, the knowledge about GP's sickness certification practice is very limited [1-3]. Sickness certification involves several elements such as assessing the degree to which the reduced functional capacity limits a patient's capacity to work, to determine the optimal grade and probable duration of sick leave, and together with the patient consider advantages and disadvantages of sick leave [4].

Studies from different countries on sickness certification have indicated that physicians, and especially GPs, experience the tasks and situations relating to sickness absence consultations as problematic [1,3,2,5-11]. In some studies, sickness certification has been characterized as a burden for the physician [12] or even as a working environment problem [11]. Aspects found to be problematic include assessment of patients' functional ability, work capacity, and the need for sickness absence [4,10,11,13-15]. In relation to sickness certification, many physicians find it problematic to handle the two roles as the treating physician and as the medical expert to the social insurance services [10-17]. Physicians also find it problematic handling situations where they and the patient disagree on the need for sick leave [4,10,11,14-17]. The role of GPs in sickness certification differs somewhat between countries in Western Europe [18]. Nevertheless, our knowledge of GPs' sickness certification practices remains limited [2].

Although a few studies have compared rates of sickness absence between countries [18-21], the findings are difficult to compare since differences in sickness insurance systems are seldom taken into account. Very few attempts have been made to compare sickness certification practices and physicians' experiences with sickness certification across countries [2,22]. Even between countries with very similar social insurance systems, such as Sweden and Norway, there are differences in both the organization of GP services and in sickness insurance systems. Such differences may influence both rates of sickness absence and GPs' experiences [23]. Comparison of data between countries might provide new insight in this research area.

## Aim

The aim of this study was to gain knowledge about GPs' experiences of sickness certification regarding frequencies of sickness certification consultations and aspects they found problematic, and to compare this between Sweden and Norway.

## Methods

We analyzed and compared data from cross-sectional surveys of GPs in Norway and in Sweden concerning their experiences with sickness certification.

### The two questionnaires

#### Sweden 2008

A comprehensive questionnaire on sickness certification was developed based on a previous study in 2004 [10], the research literature, a pilot study, and discussions with a reference group [8]. The questionnaire comprised 163 questions including frequency of sickness absence consultations, how often the physician performed various tasks and situations relating to sickness certification, and how problematic they experienced these tasks and situations [24]. The thorough work in development of the items and that it was tested in several pilot studies grant for high face validity. The questionnaire was sent out in October 2008. Three reminders were mailed to non-responders.

#### Norway 2010

Forty-six questions on sickness certification were included in a survey involving a total of 300 questions, e.g. about work environment of physicians. The survey was sent to a representative panel of physicians in Norway [25]. Of these 46 questions on sickness certification, 19 were translated into Norwegian directly from the Swedish questionnaire, three were adapted from the Swedish questionnaire to fit the Norwegian system, and 24 were new. The questionnaire was distributed in November 2010 by the Research Institute of the Norwegian Medical Association. Two reminders were sent.

### Study sample--GPs

The Swedish questionnaire was sent to all physicians working and living in Sweden (n = 36 898). In Norway, the study population comprised a representative panel of fully authorized physicians working in Norway (n = 1543). The response rates were 60.6% in Sweden and 66.5% in Norway (Table 1). In the present study, we included fully authorized and registered (that is, board certified) physicians below 68 years of age, working as GPs, and having sickness certification consultations at least a few times a year. In Norway, the sample comprised regular GPs with a contract with local authorities and participating in the Norwegian list-based GP programme.

**Table 1:** Characteristics of the Swedish and Norwegian study population, GPs in Sweden and Norway

		All participating GPs	Registered GPs < 68 years old		GPs < 68 years old with sickness certification consultations at least a few times per year^1^	
		**N (% women)**	**N (% women)**	**% of the participants**	**N (% women)**	**% of registered GPs**

**Sweden**		4394 (50)	4047 (49.5)	92.1	**3949 (49.4)**	**97.6**

**Norway**		224 (31.4)	222 (31.5)	98.7	**221 (31.4)**	**99.5**

**GP gender^2^**						

*Sweden*	Women	2196	2003	91.2	**1952**	**97.5**

	Men	2198	2044	92.9	**1997**	**97.7**

*Norway*	Women	70	70	100	**69**	**98.5**

	Men	153	152	99.3	**152**	**100.0**

**GP Age^3^**						

*Sweden*	25-52	2412 (58.0)	2144 (57.0)	88.9	**2114 (56.8)**	**98.6**

	53-67	1906 (41.1)	1903 (41.0)	99.8	**1835 (40.9)**	**96.4**

	68+	74 (18.9)	-	-	**-**	**-**

*Norway*	25-52	106 (41.5)	103 (41.5)	100.0	**106 (41.5)**	**100.0**

	53-67	116 (22.4)	116 (22.5)	100.0	**115 (21.7)**	**99.2**

	68+	1 (0.0)	-	-	-	-

^1 ^Study population of GPs in bold. ^2^Information on gender was missing for one responding GP in the Norwegian study material. ^3 ^The youngest GP in Sweden was 25 years of age, in Norway the youngest GP was 29 years

### The Swedish and Norwegian contexts

#### Social insurance and sickness absence

In both Sweden and Norway, sickness absence benefits are regarded as a universal right for residents. However, in Norway only employed persons and those receiving unemployment benefits are covered, whereas in Sweden other groups such as students and housewives can also be certified sick and receive sickness benefits if they have some income. Sweden has one qualifying day for sickness benefits, meaning that there are no sickness benefits for the first days of a new sick-leave spell. Thereafter, the sickness benefits in Sweden cover approximately 80% of lost income up to a specified limit (€29 152 in July 2011) [26]. In Norway, there is no qualifying day, with sickness benefits reimbursing 100% of income up to a limit of €62 577 (July 2011) [27]. In both countries, the first days of benefits are paid by the employer and thereafter by the National Insurance Services. A sickness certificate is required after one week of self-certification in Sweden. In Norway, half of the working population, namely those employed in companies not participating in the Agreement on a More Inclusive Working Life, have a three-day self-certification period, whereas those in participating companies have eight days. The maximum length of sick leave is one year in both countries.

#### Organization of GP services

Norway has had a list-based GP system since 2001, with approximately 98% of the population registered with a regular GP. Most Norwegian GPs are self-employed and are reimbursed partly by capitation payments and partly (70%) by a fee-for-service scheme. In Sweden, most GPs work in primary health care centres and receive a fixed salary. Residents register with a primary health care centre or a specific GP and can change GP more often than in Norway, where a change of GP is allowed twice a year or if a person moves to another municipality. In addition children of list patients can chance freely.

### Measurements

The questions on frequency of sickness absence consultations had six response alternatives: 1. never or almost never (excluded from the analyses), 2. a few times a year, 3. about once a month, 4. 1-5 times a week, 5. 6-20 times a week, and 6. more than 20 times a week.

The same response alternatives were used in both countries to measure the frequency of different tasks and situations. Thereafter, a number of questions followed regarding frequency of tasks and situations, where, in Sweden, the response alternative 5 was "6-10 times a week" and alternative 6 was "more than 10 times a week".

Another set of questions focused not on frequency as those above, but on severity, that is how problematic different tasks and situations were perceived by the GP, using the following response alternatives: 1. very problematic, 2. fairly problematic, 3. slightly problematic, and 4. not at all problematic.

### Statistical analyses

#### Descriptive analyses

PASW Statistics (SPSS) version 17 was used in the statistical analyses. Descriptive statistics were calculated for the Swedish and Norwegian study samples separately, stratified by GP gender and age (25-52 years and 53-67 years) (Tables 1 and 2).

**Table 2:** Frequency of sickness absence consultations among the GPs^1 ^in Sweden and Norway

Sickness certification consultations:		> 20 times a week	6-20 times a week	1-5 times a week	About once a month	A few times a year	All
	**N**	**%**	**%**	**%**	**%**	**%**	**%**

GPs^1^							

Sweden	3949	2.5	41.3	51.0	4.8	0.5	100

Norway	221	38.0	57.5	4.0	0.5	0	100

^1^Included were those GPs < 68 years of age, being registered physicians, and having sickness certification consultations at least a few times per year

#### Proportional differences

We used the non-parametric Mann-Whitney significance test for proportional differences between independent samples in the comparative analyses. Firstly, we compared the proportion of GPs in the two countries who had sickness absence consultations and were engaged in different tasks and situations concerning sickness absence a number of times a year (about once a month or 1-5 times a week) (Table 3). Secondly, we compared the proportion of GPs reporting that each of the different tasks and situations associated with sickness absence consultations was very or fairly problematic (Table 4). The significance level was set to *p *< 0.05 in the Mann-Whitney analyses (Tables 3 and 4).

**Table 3:** GPs engaged in tasks and situations related to sickness certification at least once a week

Proportion of GPs that at least once a week:	Sweden (n = 3949)	Norway (n = 221)	**Proportional diff^1^**.	**Mean diff^1^**.	*p *value^2^
. . . experiences patients partly or completely say no to sick leave they suggest.	9.3	9.3	1.0	0.0	0.098

. . . say no to a patient who wants a sickness certificate.	15.0	10.4	1.44	4.6	0.075

... experience conflicts with patients about sickness certification.	14.1	11.5	1.23	2.6	0.335

. . . experience patients say that they will change physician if they do not issue a sickness certificate.	2.8	5.1	0.54	-2.3	0.398

... have consultations including consideration of sickness certification.	94.9*	99.5*	0.95	-4.6	**0.002**

. . . find it problematic to handle sickness certification.	55.0*	43.3*	1.27	11.7	**0.001**

. . . encounter a patient who wants to be on sick leave for some other reason than work incapacity due to disease or injury.	27.6*	9.5*	2.9	18.1	**0.000**

. . . worry that patient will go to another physician if they do not issue a sickness certificate.	7.0*	17.5*	0.4	-10.5	**0.017**

^1^Differences between groups reported as proportional difference and mean difference

^2^*P*-values for mean difference calculated using the Mann-Whitney test

*p = < 0.01 (in bold)

**Table 4:** Proportion of GPs in Sweden and Norway, respectively, who perceived different sickness certification tasks and situations as problematic

Proportion of GPs that generally find it very problematic or fairly problematic to:	Sweden (n = 3949)	Norway (n = 221)	**Proportional diff^1^**.	**Mean diff^1^**.	*p *Value^2^
. . . assess the optimum duration and degree of sickness absence.	69.1	71.4	0.97	-2.3	0.487

. . . provide a long-term prognosis about the future work capacity of patients on sick leave.	77.3	72.3	1.07	5.0	0.087

. . . handle sickness certification of patients.	59.9*	52.5*	1.14	7.4	**0.030**

. . . assess the degree to which of the reduced functional capacity limits patient's work capacity.	80.6*	67.9*	1.19	12.7	**0.000**

... discuss with the patient the advantages and disadvantages of being on sick leave.	40.5*	31.1*	1.30	9.4	**0.005**

... manage the two roles as the patient's treating physician and a medical expert for the social insurance office and other authorities.	64.5*	52.3*	1.23	12.2	**0.000**

... decide whether to certify a prolongation of a sick-leave period initially certified by another physician.	69.5*	30.9*	2.25	38.6	**0.000**

... handle situations in which you and your patient have different opinions about the need for sickness leave.	61.1*	74.7*	0.82	-13.6	**0.000**

^1^Differences between groups reported as proportional difference and mean difference

^2^*P*-values for mean difference calculated using the Mann-Whitney test

*p = < 0.01 (in bold)

#### Logistic regression

Logistic regression was used to identify factors associated with GPs experiencing sickness certification as problematic (very or fairly). The regression analyses were performed separately for Sweden and Norway. The independent variables were frequency of sickness absence consultations, GP gender and GP age group (25-52 years and 53-67 years). The frequency of sickness absence consultations was recoded into three categories: 1. a few times a year (from once a month to at least once a week), 2. 6-20 times a week, and 3. more than 20 times a week. Group 2 was used as the reference group and the significance level was set to *p *< 0.05 in the analyses (Table 5).

**Table 5:** Factors associated with GPs experiencing problematic sickness absence consultations, in Sweden and Norway, respectively.

	Sweden			Norway		
***Independent variables***	**Odds ratio**	**95% CI**	**Coefficents**	**Odds ratio**	**95% CI**	**Coefficents**

***Frequency of sickness certification consultations***						

Sometimes each year, about once a month and 1-5 times a week	0.812*	(0.708-0.931)	-0.209	1.052	(0.680-1.626)	0.050

6-20 times a week (Ref.)	1*			1*		

> 20 times a week	1.461	(0.933-2.290)	0.379	2.303*	(1.920-2.762)	0.834

***GP gender***						

Men (Ref.)	1*			1		

Women	0.808*	(0.705-0.926)	-0.213	1.021	(0.843-1.236)	-0.020

***GP age***						

24-52	0.649*	(0.567-0.744)	-0.432	0.580*	(0.486-0.692)	-0.545

53-67 (Ref.)	1*			1*		

Logistic regression with 95% confidence intervals (CI)

R^2 ^(fully adjusted model) Sweden: 0.026; Norway: 0.075

*p = < 0.01 (in bold)

The study was approved by the Regional Ethical Review Board of Stockholm, Sweden, and the Norwegian Privacy Ombudsman for Research, Norway.

## Results

### Participants--GPs in Sweden and Norway

The study sample comprised 3949 GPs from Sweden and 221 GPs from Norway (Table 1). The proportion of women was 49% in the Swedish sample and 31% in the Norwegian sample. In Sweden, 54% of the GPs were below 53 years versus 48% in Norway.

### Frequency of sickness absence consultations, tasks and situations

In the Swedish sample, 95% of the GPs and in the Norwegian sample almost 100% of the GPs had sickness absence consultations at least once a week (Table 2). Thirty-eight percent of the GPs in Norway reported having sickness absence consultations > 20 times a week and 58% between 6 and 20 times a week. In Sweden, the corresponding figures were just 3% and 42%, respectively.

Approximately 10% of participating GPs from both countries had consultations every week where patients declined to take sick leave suggested by the GP. In both countries, 10-15% reported that they every week declined to issue a sickness certificate in spite of a patient's request (Table 3). Between 11 and 15% experienced conflicts with patients, and 3-5% every week encountered patients who stated that they would change GP if they did not receive a certificate.

A significantly larger proportion, 55% of GPs in Sweden and 43% in Norway, reported finding it problematic to handle sickness certification consultations at least once a week. Twenty-eight percent of GPs in Sweden encountered patients who wanted to be on sick leave for reasons other than work incapacity due to disease or injury. In Norway, 10% of GPs experienced such situations every week (*p *< 0.05).

However, a larger proportion of GPs in Norway (18%) than in Sweden (7%) worried at least once a week that patients would go to another physician if they did not issue a sickness certificate (*p *< 0.05).

### Predictors of problematic consultations

A higher frequency of sickness absence consultations was associated with a higher risk of GPs experiencing sickness absence consultations as problematic in both countries, adjusted for GP gender and age (Table 5).

### Frequency of problematic situations

Generally, a majority of the GPs in both countries found all the specified aspects of sickness certification problematic. For instance, 81% of the Swedish sample and 68% of the Norwegian sample found it problematic to assess the degree to which the reduced functional capacity limited a patient's capacity to work (*p *< 0.05). A similar proportion (69-71%) of GPs in Sweden and Norway found it difficult to assess the optimum duration of sick leave (Table 4).

### Other significant differences between Norway and Sweden

A significantly larger proportion of GPs in Sweden, compared to Norway, reported that they found it difficult to discuss with the patients the advantages and disadvantages of being on sick leave (41% versus 31%). A significantly larger proportion of GPs in Sweden also found it problematic to manage the two roles of being the patient's treating physician and medical expert to the social insurance services (65% versus 52%). This was also the case when considering whether to issue a prolongation of a sick-leave period initially certified by another physician (70% versus 31%).

However, a significantly larger proportion of GPs in Norway (75%) reported finding it problematic to handle situations in which they and their patient disagreed on the need for sick leave. The corresponding figure for Sweden was 61%.

## Discussion

In this large survey, nearly all the participating GPs had sickness absence consultations at least once a week. However, 95% of the GPs in Norway had six or more such consultations every week compared to 44% in Sweden. A large majority of the GPs in both countries experienced sickness certification generally as very or fairly problematic. Indeed, a majority of the GPs found most of the examined aspects of sickness absence consultations problematic.

Some of the aspects of sickness certification were experienced as problematic for more of the GPs in Sweden than GPs in Norway. This included discussing the advantages and disadvantages of being sickness absent and to issue a prolongation of a sick-leave period initiated by another physician. Ten percent of the Norwegian GPs and 28% of the Swedish GPs reported encountering patients each week that requested sick leave for reasons other than disease or injury.

GPs in Norway worried more often than Swedish GPs that patients would go to another physician if they did not issue a certificate. A significantly larger proportion of GPs in Norway also found it problematic to handle situations in which they and their patient disagreed on the need for sick leave.

### Strengths and limitations

To our knowledge, this is the so far largest study comparing GPs' experiences of sickness certification. Furthermore, this is the first survey study in which a large number of physicians in two countries have been asked several identical questions on sickness certification experiences. Three main strengths of this study are thus the size of the study groups, that GPs from two countries were included, and the many similar questions. The questions used in the studies were subject to discussion among researchers and in pilot-studies in both countries. In addition an effort were made making the two study materials from Sweden and Norway match each other as far as possible (regarding the GPs level of education, and the gender and age distribution). In Sweden, the study population comprised all physicians in the country, and in Norway a representative sample of physicians were invited to participate. A further strength is that the same questions were used in a previous study in 2004 in Sweden [10], and the questions had been thoroughly evaluated in both countries, undergoing revisions based on feedback from researchers and physicians. The response rates were acceptable, 60.6% in Sweden and 66.5% in Norway.

A possible limitation is the fact that the surveys in Sweden and Norway were somewhat different. In Sweden all questions were related to sickness certification while the scope of the Norwegian survey was more comprehensive, including questions on other topics, e.g. the work environment of physicians. This might have lead to differences in the participants, e.g. have lead to those GPs with special interest in sickness certification aspects choose to answer in Sweden but not in Norway.

One general limitation of survey studies is that informants might interpret questions in different ways and that they, regarding frequencies, provide only self-reported information, which may differ from actual practice [28]. Respondents in survey studies also have a general tendency to give positive answers to questions [29,30], for example understating the frequency of problems they encounter. However, we have no reason to believe that GPs in either country would be more accurate or have less recall bias.

### Problems experienced by GPs

This study confirms results from several studies, that sickness certification is experienced as problematic by physicians, especially by GPs [1,4,2-7,9-11,13,16,17,31]. The study also shows that there are differences between countries regarding GPs' experiences of sickness certification.

### The two roles

GPs have two roles in sickness certification, one as a treating physician and the other as a medical expert providing accurate medical information to the social insurance services. In the present study, more than 50% of the GPs in both countries felt that managing these two roles was problematic. This agrees with other studies [10,12-15,17,32,33]. Clearly, this calls for interventions and support regarding both training and health care management [1,34,35].

### Non-medical reasons

The qualification requirements for sickness benefit--reduced work capacity due to injury or disease--are the same in both countries. However, three times as many GPs in the Swedish sample--28% versus 10%--each week encountered patients that wanted to be on sick leave for a non-medical reason. Moreover, GPs in Sweden generally experienced sickness certification tasks as more problematic than did their colleagues in Norway. One possible explanation for these differences is that GPs in Norway often see patients at an earlier stage in the sickness absence process, as half of the working population need a sickness certificate already after three days of self-certification--most sick-leave spells do not last more than a week. Another possible explanation is that patients at risk for sick leave differ to some extent between the two countries. A larger proportion of the population in Sweden is eligible for sickness benefits, meaning that expectations of sickness certification might differ. GPs in Sweden and Norway might also differ in how they define the concept of disease. Many Norwegian GPs tend to define conditions such as grief or illness of a spouse as a disease [12]. Nevertheless, the proportion of GPs in both countries that regularly declined to issue sickness certificates to patients was very similar (10-15%).

### Conflicts with patients

Actually, one out of ten GPs in both countries experienced conflicts with patients about sickness certification as often as every week. Previous studies have found that conflicts with patients and other stakeholders are a common problem for physicians [3], and some GPs experience the task of sickness certification as so problematic that it was deemed a working environment issue [11]. The proportion of GPs in this study experiencing frequent conflicts with patients could be regarded as high, viewed in the perspective of the GPs' working environment. This may affect GPs in different ways and warrants interventions. Previous studies have indicated that physicians ask for support and more knowledge and skills in sickness certification [12,34].

### Concern that patients will change GP

GPs in both countries worried that patents would go to another physician if denied a sickness certificate. However, a significantly higher proportion of Norwegian GPs were concerned about this: 18% versus 7% in Sweden. In Norway, GPs are partly reimbursed according to the number of patients on their list. This may explain that a higher proportion of Norwegian GPs worried that patients will go to, and possibly permanently switch to, another GP if they do not receive a certificate. Thus, economic incentives in the Norwegian system may influence GPs' sickness certification practices. Previous studies have found that Norwegian GPs experience rationing decisions as difficult, especially when related to economic incentives [36,37]. This might also be relevant in sickness certification.

### Problematic experiences and certificates issued by GPs

Previous studies have found that GPs in Sweden issue 40% of all sickness certificates in the country, compared to 70-80% in Norway [38,39]. The remaining certificates are issued by hospital physicians, occupational health physicians, private specialist clinics, and out-of-hours services The stricter referral system in Norway might be one reason for this difference. The economic incentives in the Norwegian system may also influence Norwegian GPs to have more frequent consultations with their patients, including patients who are certified sick. Closer follow-up and knowledge of each patient might facilitate the negotiations between physician and patient regarding sickness absence and thereby explain why Norwegian GPs apparently experience sickness absence consultations as less problematic than their Swedish colleagues.

## Conclusions

The study confirms that sickness absence consultations, both in general and regarding specific tasks, are experienced as problematic by a majority of GPs. In spite of the many similarities between the social insurance systems of Sweden and Norway, several differences were found regarding the frequency of sickness certification consultation and to what extent different tasks and situations were experienced as problematic. Cross-country differences in GPs' experiences and the causes and consequences of these differences should be addressed in further studies.

## Competing interests

The authors declare that they have no competing interests.

## Authors' contributions

LDW is the main author of the manuscript and participated in the data collection in Norway, performed the statistical analyses and drafted the manuscript. SG and BC participated in the planning of the study, data collection in Norway and writing of the manuscript. KA and ALW participated in data collection in Sweden. KA, LK and ALW participated in the planning of the study and the writing of the manuscript. LK and ALW assisted with the statistical analyses. The final version of the manuscript has been read and approved by all authors.

## Pre-publication history

The pre-publication history for this paper can be accessed here:

http://www.biomedcentral.com/1471-2296/13/10/prepub
